# Autism under the umbrella of ESSENCE

**DOI:** 10.3389/fpsyt.2023.1002228

**Published:** 2023-01-23

**Authors:** Elisabeth Fernell, Christopher Gillberg

**Affiliations:** Gillberg Neuropsychiatry Centre, Sahlgrenska Academy, Gothenburg University, Gothenburg, Sweden

**Keywords:** autism spectrum disorder, comorbidity, early symptomatic syndromes eliciting neurodevelopmental clinical examinations (ESSENCE), attention-deficit/hyperactivity disorder, intellectual disability, etiology, intervention

## Abstract

This brief article gives a short overview of “comorbidity” in autism. The most common co-occurring disorders will be presented and discussed within the context of ESSENCE (Early Symptomatic Syndromes Eliciting Neurodevelopmental Clinical Examinations), a concept that provides a holistic perspective for neurodevelopmental disorders. The ESSENCE concept also considers the heterogeneous and changing clinical panorama of developmental disorders over time, and also the multifactorial etiologies, including so called behavioral phenotype syndromes. Aspects on behavioral interventions in autism are presented—interventions that need to be adapted and take into account all non-autism associated ESSENCE, including intellectual disability and Attention-Deficit/Hyperactivity Disorder (ADHD). The article also focuses on current research on pharmacological intervention based on the hypothesis of imbalance in excitatory/inhibitory transmitter systems in autism and some other ESSENCE.

## Introduction

Neurodevelopmental disorders (NDDs) (1, 2) is a broad term encompassing several early onset disorders with origin in the central nervous system (CNS), usually with a chronic course, and with impairments generally lasting into adulthood.

NDDs can be classified according to the *main neurological/psychiatric/behavioral functions* affected, such as motor disorders [cerebral palsy, developmental coordination disorder (DCD)], cognitive, executive function, and communication disorders [intellectual disability (ID), autism spectrum disorder (ASD), henceforth referred to as “autism”, attention-deficit/hyperactivity disorder (ADHD), and language disorder (LD)], and paroxysmal disorders (for example epilepsy).

They can also be grouped with regard to their *definite or assumed period of origin*, i.e., prenatal (before birth), perinatal (from birth to the completion of the first week or the 28th day of life) or postnatal (the period from birth up to the age of 1 year, or sometimes 2 years of age). Among the perinatally acquired disorders, two main groups encompass conditions in children born extremely preterm and in children born at term suffering hypoxic-ischaemic encephalopathy.

Yet another classification can be based on *specific identified etiologies*, including genetic/chromosomal abnormalities, a number of neurometabolic disorders, and acquired prenatal conditions, such as fetal alcohol spectrum disorder (FASD), and fetal valproate syndrome. Additional possible classifications include those based on *presumed affected brain areas*, and *types of neural networks/circuits/transmitter system aberrations in the CNS*.

## Neurodevelopmental disorders

Although, the term “neurodevelopmental disorders” has a long history, it had not been included in previous editions of either the International Classification of Diseases (ICD) or the Diagnostic and Statistical Manual of Mental Disorders (DSM) (3).

According to the ICD-11 (2), NDDs are grouped within the section: “Mental, behavioral and neurodevelopmental disorders”. “Neurodevelopmental disorders” include (i) disorders of intellectual development, (ii) developmental speech or language disorders, (iii) autism, (iv) developmental learning disorders, (v) developmental motor coordination disorder, (vi) ADHD, (vii) stereotyped movement disorder, and (viii) a category labeled “other neurodevelopmental disorders”.

NDDs according to the DSM-5 (1) include the main categories (i) Intellectual disabilities, (ii) Communication disorders, (iii) Autism spectrum disorder, (iv) Attention-deficit/hyperactivity disorder, (v) Specific learning disorder, (vi) Motor disorders and (vii) Other neurodevelopmental disorders; with defined subcategories.

## ESSENCE

The term ESSENCE, Early Symptomatic Syndromes Eliciting Neurodevelopmental Clinical Examinations, was coined by Gillberg (4). ESSENCE captures the early manifestations of NDDs that sometimes are unspecific, affecting motor, cognitive, communicative and social development, as well as sleep, feeding and temperament/behavioral regulation. The ESSENCE concept also emphasizes the very high rate of “comorbidities” and the changing presentations during early childhood and adolescence. The concept highlights signs of unspecific developmental deviations or delays, e.g., regarding speech and language and motor development. The importance of clinical follow-up to evaluate outcome and provide best-evidenced interventions is also underscored.

The ESSENCE concept includes the temporal and dynamic changes of developmental symptoms; early unspecific symptoms may become more evident and chiseled-out during the preschool and school years and later accord with a certain developmental diagnosis, a “named disorder”. ESSENCE underscores that a first recognized symptom or identified disorder need to be followed over time in a holistic perspective, taking into account all possible developmental/behavioral problems, and not over-focusing on one specific disorder only. With an “ESSENCE perspective in mind” the risk of diagnostic overshadowing, i.e., overlooking co-occurring problems in a child with e.g., autism or ADHD is minimized.

The ESSENCE concept highlights the variety of etiologies and emphasizes e.g., that the so called behavioral phenotype syndromes (BPS), very often present with neurodevelopmental/ESSENCE symptoms rather than as a physical phenotype/known genetic/medical syndrome. A common clinical scenario is a child later presenting a clear clinical picture of autism, or intellectual disability or ADHD (or combinations of these) with a first symptom/diagnosis being speech- and language delay or LD.

The ESSENCE umbrella also covers the many neurodevelopmental/neuropsychiatric symptoms/disorders that accompany traditionally defined neurological diagnoses, including epilepsy and cerebral palsy (see below).

## Language disorder, autism and ESSENCE

A common first symptom or problem in young children, later diagnosed with a specific disorder within ESSENCE, is speech and language delay. The prevalence of language delay at the Child Health Center (CHC) screening at 2.5 years is about 10% and the prevalence of a diagnosed LD in Sweden is around 6% (5).

In a study by Miniscalco et al. (6), children who had screened positive for speech and language problems at age 30 months at their CHC were followed up and examined at age 6 and 7 years. The study revealed that children in the general population who screen positive for speech and language problems before age 3 years are at very high risk of autism or ADHD, or both, at 7 years of age. Remaining language problems at age 6 years strongly predicted the presence of neuropsychiatric or neurodevelopmental disorders at age 7 years.

Over the past 20 years, there has been increasing awareness of speech and language delay as a marker of many other NDDs/ESSENCE, e.g., ID, ADHD and autism. In a multidisciplinary study, investigating children attending preschool units—specifically aimed for children with language impairments, without any other diagnosed developmental disorders before referral—revealed that about 90% had additional developmental disorders (7). A follow-up of these children 10 years later showed that a large number had persistent language problems and/or met criteria for other NDDs or had subthreshold diagnostic symptoms; mild intellectual disability, borderline intellectual functioning, autism or “autistic traits”, ADHD or subthreshold ADHD and a large number had dyslexia (8).

In another follow-up study with the aim of analyzing the further development of children, 5 years after they had screened positive for LD and/or autism at 2.5 years, clinical registers covering all relevant outpatient clinics, were reviewed with regard to registered ICD-diagnoses. The study revealed that 40% of the cohort had remaining or other developmental problems at this follow-up. It was discussed that this rate most likely was a minimum frequency and that it was expected that more children would be referred for developmental problems later on (9).

Obviously, language delays/disorders in young children are often markers for other, later diagnosed NDDs, including ID, autism and ADHD; hence, teams responsible for assessments of children with language problems need to have a broad ESSENCE approach in order to provide best possible assessment and recommendation for interventions.

The ESSENCE panorama extends beyond early childhood. Children with LD will very likely experience difficulties in learning to read, and poor language may be a common risk factor for both reading disorder and mathematics disorder (10). Schoolchildren with autism may also exhibit comprehension difficulties and problems to access and select meanings of ambiguous words which compromise their language comprehension (11).

## Autism, ADHD, ID, other neurodevelopmental and psychiatric comorbidities and ESSENCE

Comorbidities are extremely common in children with autism at all levels of intellectual functioning and language delay and language disorder are common predecessors of autism with and without associated ID, and with and without other ESSENCE.

In a meta-analysis (12), psychiatric comorbidity in children and adolescents with autism was studied with regard to ID, ADHD, anxiety disorders, sleep disorder, disruptive behaviors, bipolar disorder, depression, obsessive compulsive disorder and psychosis. The highest prevalence estimates were found for ADHD 26.2%, ID, 22.9% and anxiety disorders 11.1%. Conclusions from the study were that the frequency of psychiatric comorbidity in children and adolescents with autism is considerable and that there is a need for better targeted diagnostic tools to detect psychiatric comorbidity in children, youth, as well as adults with autism. The authors emphasized that this represents a major gap comparted to the time and careful attention given to diagnostic accuracy of autism itself. Young children (under age 5 years) with autism almost invariably have major “other/non-ASD problems” (13).

Comorbid ESSENCE was analyzed in a population-based group of more than 200 children with diagnosed autism between the ages of 20–54 months, referred to an autism center for early intervention (14, 15). The children's developmental profiles were assessed at start of intervention and after 2 years. The children were assessed in great depth by a research clinical neurodevelopmental team consisting of physicians, psychologists and speech and language pathologists. Regarding motor development, “only” 66% of the children had started to walk unsupported before age 15 months, and 23 and 11%, respectively had started between 15 and 18 months or after 18 months of age. Children in the autism group demonstrated a marked delay in the development of expressive vocabulary. The general cognitive level was crucial in this respect. At the time of the first assessment, 13% of the children had no words at all, 33% had a few single words and 54% either had a few communicative sentences or had some phrase speech with or without echolalia. ADHD was not diagnosed at this early age but 42% of the children were classified by their parents—and confirmed by the examining physician—as definitely hyperactive, 8% had an activity that was alternating between hyper- and hypoactivity, 3% were reported to be hypoactive, and 47% had an activity level within the normal variation (14). At the 2-year follow-up at ages between 4 and 6.5 years, half the group had clear ID in addition to autism and about 25% had borderline intellectual functioning, and 25% average intellectual functioning, respectively (15).

## Autism, DCD and other ESSENCE

Motor and language impairments are common and closely related in young children with autism (16). In a study of schoolchildren with autism, assessments with a motor performance test and parental reports of the child's motor and language skills revealed that 85% had motor and/or structural language deficits in addition to their social impairment. A conclusion from the study was that co-occurring motor and structural language deficits should be anticipated and assessed in the evaluation process of children with autism. Such assessment can provide a basis for specific interventions that will complement those targeting social skills deficits and other autism core symptoms (17). Children with ADHD and “comorbid” DCD often have autistic traits, whereas children with ADHD, without DCD, often have major oppositional defiant behavior/disorders (ODD) (16).

## Autism, epilepsy and other ESSENCE

Epilepsy is a common comorbidity in autism, occurring at increasingly higher rates with increasing age and is strongly linked to the co-occurrence of ID (18, 19). In some children with autism and epilepsy, the first manifestation has been infantile spasms. This variant of early onset epilepsy has in some cases proved to be linked to specific syndromes, such as tuberous sclerosis, neurofibromatosis type 1 and Down syndrome, all now known to have a strong association with epilepsy (20). The co-occurrence of epilepsy and other ESSENCE has been extensively studied (21). Prevalence rates of autism, ADHD and behavior difficulties in young children with epilepsy and in children without epilepsy showed that of those with epilepsy, 18% had autism, 40% hade ADHD and about 75% had behavioral difficulties. Thus, young children with epilepsy had a very high level of parent reported behavioral difficulties and a high risk for ADHD and autism, highlighting the need for comprehensive multidisciplinary assessments. Behavioral concerns were not greater than for other children with non-epilepsy related neurodisabilities with the exceptions regarding attention and mood. Epilepsy-related factors were not associated with child behavior, suggesting that seizures *per se* do not confer a unique risk for behavioral difficulties. The importance of early recognition of social deficits in children with epilepsy is an important aspect of the comprehensive management of this patient group (18, 19).

## Regressive autism and catatonia

Most children with autism have shown mild or marked developmental problems from early childhood (20). However, in a subgroup of about 20% there is a reported developmental regression after a typical or a marginally delayed development extending to about 18–24 (30) months of age (22–24). Children with a regressive developmental trajectory, with or without autism, always need a careful neuropediatric work-up to investigate possible neurological diseases that may lead to developmental regression, taking into account possible treatable conditions (23).

A marked functional decline or a late regression with symptoms according with catatonia can occur in adolescents with autism after a relatively stable childhood. Common symptoms are obsessive and compulsive rituals, speech regression, motor abnormalities including posturing, aggression and mood disturbance. Catatonia has been reported to be a common cause of late regression in individuals with autism (25). Although the etiology is unknown, disrupted gamma-aminobutyric acid has been proposed as the underlying pathophysiological mechanism. Key symptoms can be identified under 3 clinical domains: motor, speech, and behavior. Benzodiazepines and electroconvulsive therapy are the only known effective treatments (26).

## Autism, cerebral palsy and ESSENCE

**A** total population of school-age children with cerebral palsy were assessed with regard to the rates of autism and ADHD and the relationships between these disorders and motor function, ID, and other associated impairments. The study showed that 45% of the children met criteria for autism, ADHD, or both. ID was present in 51%. Two-thirds had autism, ADHD, and/or ID. It was concluded that autism and ADHD were common in this population of children with cerebral palsy and mainly independent of motor severity and cerebral palsy type. The strongest predictor of autism/ADHD was ID. Assessment for autism and ADHD is warranted as part of the evaluation in cerebral palsy in both term and preterm born children (27, 28).

## Autism, ESSENCE and underlying etiologies, including behavioral phenotype syndromes

Every young person diagnosed with a neurodevelopmental disorder needs a medical evaluation. In children without specific complications indicating perinatal or postnatal adverse events, the etiology is most likely prenatal. Among the prenatal etiologies, there are chromosomal/genetic as well as acquired causes. A detailed history from parents, supplemented with data from records and an examination of the child will provide guidance for specific medical examinations. In children with autism, a specific medical/etiological diagnosis will be clinically established in about 20% of the cases and is more often identified when the child has concomitant ID (29).

There are several sex chromosome trisomies—XXX, XXY, and XYY—that are associated with autism (30). The most well-known syndrome due to an autosomal trisomy is Down syndrome, which is the most common, identified single cause of ID. Down syndrome, occurs in about 1/800 newborns. In 2001, Rasmussen et al. reported autistic disorders in Down syndrome (31). In a recent population-based study, 42% of children with DS also met criteria for autism. An important minority (34%) met criteria for ADHD (32).

There are many other behavioral phenotype syndromes with identified prenatal etiologies, mostly genetic but also prenatally acquired. Genetically defined behavioral phenotype syndromes include syndromes that have an identified genetic mutation; FragileX syndrome, Rett syndrome, Tuberous sclerosis and Neurofibromatosis type 1, to mention a few conditions for which the ESSENCE symptoms may precede the diagnosis of the genetic disorder (13).

Through extensive genetic research, a number of causes of neurodevelopmental disorders, including autism and other ESSENCE have been clarified and more than 1,500 genes associated with conditions such as ID and autism have been identified (33). These NDD genes are distributed over all chromosomes on autosomes, on the X chromosome, and a few on the Y chromosome and on the mitochondrial genome (33).

ESSENCE symptoms always need to be evaluated with regard to etiological and pathogenetic factors. Among these, genetic factors predominate. The heritability and genetic architecture of autism is complex and the genetic risk for autism is shaped by a combination of rare and common variants and thus the genetic susceptibility to autism can vary from one individual to another (34).

In a Swedish study, parents of all 9- and 12-year-old twin pairs born between 1992 and 2000 were interviewed regarding autism spectrum disorders and associated ESSENCE. Concordance rates and structural equation modeling were used for evaluating causes for familial aggregation and overlap across diagnostic conditions. A high comorbidity was found across the different ESSENCE neuropsychiatric disorders, and the data suggest that genetic effects are of major importance for this comorbidity (35).

Genetic disorders are variably expressive, in that the children with the same variant may show severe features while carrier parents show mild features. For example, in the assessment procedure of a boy with specific ESSENCE symptoms, including autism, an underlying condition may be an inherited Fragile X condition with a full mutation, while the mother has a premutation with no or a very mild cognitive/executive dysfunction (36).

By using chromosomal microarray analysis, a high resolution chromosomal technique to detect submicroscopic chromosomal rearrangements smaller than 100 kb, an increased prevalence of copy number variants (CNVs) and single nucleotide variants (SNVs), affecting genes, have been reported in patients with autism. Several recurrent CNVs have been associated with autism, reaching genome-wide significance, such as duplications at 15q11-13, deletions/duplications at 16p11.2 and 22q11.2 and 11q24.2-25 (37, 38).

There are several syndromes related to copy number variants, such as deletions and duplications. The 22q11 deletion syndrome occurs in about $\frac{1}{4}$,000 newborns and gives rise to many symptoms of varying severity. There is a high incidence of cardiac malformations, cleft palate, velopharyngeal insufficiency and immune deficiency due to hypoplasia or aplasia of the thymus. The syndrome can be inherited or occur as a new deletion. Cognitive symptoms within ESSENCE are common and mostly relatively mild; mainly autistic features, ADHD and mild intellectual disability (39).

Among the prenatally acquired syndromes, fetal alcohol spectrum is the most common and related to the fetus' alcohol exposure (40, 41). Common ESSENCE symptoms in these children are ADHD, mild intellectual disability and autism. Genetic aspects, e.g., ADHD-heredity, may also be involved.

An association between congenital hypothyroidism/hypothyreosis, i.e., thyroid hormone deficiency present at birth, and autism was reported 30 years ago (42). Newborn screening programs have led to earlier diagnosis and treatment, resulting in improved neurodevelopmental outcomes (43).

Another prenatal acquired syndrome that may cause autism and other ESSENCE is caused by a cytomegalovirus infection (CMV). The infection can be identified through analysis of the CMV DNA from the dried blood spots from the newborn metabolic screening. Congenital CMV is one of the many etiologies underlying autism and a rate of 3% of congenital CMV has been found in children with autism with intellectual disability (44).

Warrier et al. (45) have highlighted the considerable phenotypic heterogeneity in autism and emphasized that deeper phenotypic characterization will be critical in determining how the complex underlying genetics shape cognition, behavior and co-occurring conditions in autism.

Extremely preterm birth (i.e., birth occurring before a gestational age of 27–28 weeks) infers a much increased risk for ESSENCE. Follow-up studies conducted in the preschool years, school age and adolescence, and adulthood point to an increased risk for inattention, socio-communicative problems and emotional difficulties in individuals born extremely preterm (46).

In a follow-up of children born before a gestational age of 24 weeks, 75% had neurodevelopmental disorders, including speech disorders (52%), ID (40%), ADHD (30%), autism (24%), visual impairment (22%), cerebral palsy (17%), epilepsy (10%) and hearing impairment (5%). The majority also had other specific medical diseases, such as asthma (63%) and failure to thrive/short stature (39%) (47).

## Autism and behavioral intervention, the role of ESSENCE

In a systematic review by Howlin and her group of early behavioral interventions for children with autism, the authors formulated a conclusion that fits well with the concept of ESSENCE: “Assessing what treatments work for which children and identifying the individual characteristics that predict responsiveness to specific programs and approaches, are the challenges that lie ahead (48). The latest Cochrane systematic review of Early intensive behavioral intervention (EIBI) for young children with autism (49) emphasized that early intensive behavioral intervention (EIBI) is one of the more frequently used interventions, delivered for many years for autism, often at an intensity of 20–40 h per week, and based on the principles of applied behavior analysis (ABA). Conclusions from this Cochrane systematic review were that there is only weak evidence that EIBI may be an effective behavioral treatment for *some* children with autism and that additional studies using rigorous research designs are needed to make stronger conclusions about the effects of EIBI for children with autism. Among implications for research, the authors mentioned that individuals with autism are diverse in their symptom presentation and vary greatly in cognitive functioning level (for example, from severe intellectual disability to well-above average intelligence) and that comparative effectiveness studies are needed to determine if EIBI is more effective than other active treatments recommended for children with autism.

The conclusions from the Cochrane review were consistent with those by Frans et al. (50) discussing that while many early interventional approaches have an impact on child outcomes, study heterogeneity and quality had an impact on our ability to draw firm conclusions regarding which treatments are most effective.

In a study of clinical predictors for outcome of behavioral interventions in children with autism, the child's general intellectual level was the most important single predictor. Cognitive level at start of intervention (dichotomized into IQ < 70 and IQ≥70) made a unique and statistically significant contribution to outcome prediction. The findings have significant clinical implications in terms of prognostic information given to parents at the time of clinical diagnosis and when planning intervention for preschool children with autism (51).

Early interventions in children with autism need an individual approach, focusing on improving the child's communicative abilities, social interaction and everyday functioning and measures have to consider the child's total clinical presentation, beyond autism and include also other problems/disorders under the ESSENCE umbrella. In a prospective, naturalistic study of more than 200 young children, half of whom with ID, effects on adaptive functioning of early intervention—intensive or non-intensive—were analyzed after 2 years. It was found that there was no significant difference between the intensive and non-intensive groups. The data did not support that children with autism generally benefit more from the most intensive ABA intervention programs than from less intensive interventions or targeted interventions based on ABA. Other ESSENCE, especially ID, need to be considered in the intervention planning approach (15).

## Autism and pharmacological treatment

There is no Food and Drug Administration (FDA) approved pharmacological treatment for the core symptoms of ASD. Risperidone, a second-generation antipsychotic, was the first drug approved by the FDA to treat autism-related irritability and aggressiveness (52).

Research from both animal autism models and human subjects indicates that deficits in GABAergic signaling, may contribute to the symptoms found in patients with autism (53, 54). The mechanism is related to higher chloride levels in immature neurons, leading to paradoxical excitatory actions of GABA (55). Bumetanide a selective NKCC1 chloride importer antagonist, has been reported to alter synaptic excitation-inhibition (E-I) balance by potentiating the action of γ-aminobutyric acid (GABA), thereby attenuating the severity of autism symptoms in animal models (56). The first study with bumetanide in children with autism included five children and showed significant improvements on core autistic symptoms (55). Following randomized controlled trials, including larger patient groups have also reported improvements of core autism symptoms (56–58). However, the study by Sprengers et al. (59) did not show an effect on the primary outcome of broad autism symptomatology, but suggest efficacy of bumetanide on the secondary outcome measure, repetitive behaviors in a subset of patients. These findings highlight the complexity of autism heterogeneity in trial research and the necessity of inclusion of functional brain measures to understand treatment effect variability and to develop stratification markers (59).

## ESSENCE in adulthood

Most disorders under the ESENCE umbrella persist into adulthood, but for some individuals full symptom criteria are no longer met in adult life. On the other hand, not all children with an ESSENCE disorder are diagnosed during childhood and individuals diagnosed with for example anxiety and depression as adults may have underlying ESSECE disorders, such as ADHD and/or autism. Thus, an underlying ESSENCE disorder is common among patients in adult psychiatry and should always be considered (13).

## Summary and conclusion

NDDs/ESSENCE of different types are common (affecting at least one in ten of all children), occur mostly in combinations, have different severities, numerous etiologies, and have effects on outcome during childhood and adolescence (and adulthood). Early symptoms in children with autism may be related to motor development, speech, language and communication and to regulatory problems, including sleep, feeding and emotional regulation. All levels of co-occurring ID have an impact on outcome in children with autism, receiving early intensive behavioral intervention. Some “comorbid” disorders/problems are not always evident before school age, but will become evident later during the school years. Identification, assessment in a multidisciplinary team, including a medical work-up, adapted interventions, parental psycho-educational support and follow-up through childhood and adolescence are key aspects for the child's overall development and health.

## Data availability statement

The original contributions presented in the study are included in the article/supplementary material, further inquiries can be directed to the corresponding author.

## Author contributions

All authors listed have made a substantial, direct, and intellectual contribution to the work and approved it for publication.

## References

[1] American Psychiatric Association (APA). Diagnostic and Statistical Manual of Mental Disorders, 5th edition, DSM-5. Washington, DC: APA (2013). 10.1176/appi.books.9780890425596

[2] World Health Organization. International Statistical Classification of Diseases and Related Health Problems (11th Revision). Geneva: WHO (2018).

[3] Stein DJ Szatmari P Gaebel W Berk M Vieta E Maj M. Mental, behavioral and neurodevelopmental disorders in the ICD-11: an international perspective on key changes and controversies. BMC Med. (2020) 18:21. 10.1186/s12916-020-1495-2 31983345 PMC6983973

[4] Gillberg C. The ESSENCE in child psychiatry: early symptomatic syndromes eliciting neurodevelopmental clinical examinations. Res Dev Disabil. (2010) 31:1543–51. 10.1016/j.ridd.2010.06.002 20634041

[5] Miniscalco C. Language Impairment in Swedish Children—A Survey of 6-Year-Olds Screened for Developmental Languagedisability at 2.5 and 4 years of Age. (Thesis). Göteborg University, Sweden, (2003).

[6] Miniscalco C Nygren G Hagberg B Kadesjö B Gillberg C. Neuropsychiatric and neurodevelopmental outcome of children at age 6 and 7 years who screened positive for language problems at 30 months. Dev Med Child Neurol. (2006) 48:361–6. 10.1017/S0012162206000788 16608544

[7] Fernell E Norrelgen F Bozkurt I Hellberg G Löwing K. Developmental profiles and auditory perception in 25 children attending special preschools for language-impaired children. Acta Pædiatrica. (2002) 91:1108–15. 10.1111/j.1651-2227.2002.tb00107.x 12434898

[8] Ek U Norrelgen F Westerlund J Dahlman A Hultby E Fernell E. Teenage outcomes after speech and language impairment at preschool age. Neuropsychiatr Dis Treat. (2012) 8:221–7. 10.2147/NDT.S30106 22701322 PMC3373203

[9] Miniscalco C Fernell E Thompson L Sandberg E Kadesjö B Gillberg C. Development problems were common five years after positive screening for language disorders and, or, autism at 2.5 years of age. Acta Paediatr. (2018) 107:1739–49. 10.1111/apa.14358 29637606

[10] Snowling MJ Moll K Hulme C. Language difficulties are a shared risk factor for both reading disorder and mathematics disorder. J Exp Child Psychol. (2021) 202:105009. 10.1016/j.jecp.2020.105009 33126134 PMC7677889

[11] Henderson LM Clarke PJ Snowling MJ. Accessing and selecting word meaning in autism spectrum disorder. J Child Psychol Psychiatry. (2011) 52:964–73. 10.1111/j.1469-7610.2011.02393.x 21401594

[12] Mutluer T Aslan Genç H Özcan Morey A Yapici Eser H Ertinmaz B Can M. Population-based psychiatric comorbidity in children and adolescents with autism spectrum disorder: a meta-analysis. Front Psychiatry. (2022) 13:856208. 10.3389/fpsyt.2022.856208 35693977 PMC9186340

[13] Gillberg C. The Essence of Autism and Other Neurodevelopmental Conditions. Rethinking co-Morbidities, Assessment, and Intervention. London and Philadelphia: Jessica Kingsley Publishers (2021).

[14] Fernell E Hedvall A Norrelgen F Eriksson M Höglund-Carlsson L Barnevik-Olsson M. Developmental profiles in preschool children with autism spectrum disorders referred for intervention. Res Dev Disabil. (2010) 31:790–9. 10.1016/j.ridd.2010.02.003 20207104

[15] Fernell E Hedvall Å Westerlund J Höglund Carlsson L Eriksson M Barnevik Olsson M Holm A Norrelgen F Kjellmer L Gillberg C. Early intervention in 208 Swedish preschoolers with autism spectrum disorder. A prospective naturalistic study. Res Dev Disabil. (2011) 32:2092–101. 10.1016/j.ridd.2011.08.002 21985993

[16] Gillberg C Kadesjö B. Why bother about clumsiness? The implications of having developmental coordination disorder (DCD). Neural Plast. (2003) 10:59–68. 10.1155/NP.2003.59 14640308 PMC2565425

[17] Reindal L Nærland T Sund AM Glimsdal BA Andreassen OA Weidle B. The co-occurrence of motor and language impairments in children evaluated for autism spectrum disorder. An explorative study from Norway. Res Dev Disabil. (2022) 127:104256. 10.1016/j.ridd.2022.104256 35580394

[18] Danielsson S Gillberg IC Billstedt E Gillberg C Olsson I. Epilepsy in young adults with autism: a prospective population-based follow-up study of 120 individuals diagnosed in childhood. Epilepsia. (2005) 46:918–23. 10.1111/j.1528-1167.2005.57504.x 15946331

[19] Tuchman R. What is the relationship between autism spectrum disorders and epilepsy? Semin Pediatr Neurol. (2017) 24:292–300. 10.1016/j.spen.2017.10.004 29249509

[20] Coleman M Gillberg C. The Autisms. Oxford: Oxford University Press (2012). 10.1093/med/9780199732128.001.0001

[21] Reilly C Atkinson P Memon A Jones C Dabydeen L Helen Cross J. Autism, ADHD and parent-reported behavioral difficulties in young children with epilepsy. Seizure. (2019) 71:233–9. 10.1016/j.seizure.2019.08.003 31425870

[22] Tuchman R. Autism. Neurol Clin. (2003) 21:915–32. 10.1016/S0733-8619(03)00011-2 14743656

[23] Baird G Charman T Pickles A Chandler S Loucas T Meldrum D. Regression, developmental trajectory and associated problems in disorders in the autism spectrum: The SNAP study. J Autism Dev Disord. (2008) 38:1827–36. 10.1007/s10803-008-0571-9 18449635

[24] Thompson L Gillberg C Landberg S Kantzer AK Miniscalco C Barnevik Olsson M. Autism with and without regression: a two-year prospective longitudinal study in two population-derived Swedish cohorts. J Autism Dev Disord. (2019) 49:2281–90. 10.1007/s10803-018-03871-4 30734177 PMC6546868

[25] Ghaziuddin M. Catatonia: a common cause of late regression in autism. Front Psychiatry. (2021) 12:674009. 10.3389/fpsyt.2021.674009 34777033 PMC8585308

[26] Ghaziuddin N Andersen L Ghaziuddin M. Catatonia in patients with autism spectrum disorder. Psychiatr Clin North Am. (2021) 44:11–22. 10.1016/j.psc.2020.11.002 33526232

[27] Påhlman M Gillberg C Himmelmann K. Autism and attention-deficit/hyperactivity disorder in children with cerebral palsy: high prevalence rates in a population-based study. Dev Med Child Neurol. (2021) 63:320–7. 10.1111/dmcn.14736 33206380 PMC7894137

[28] Hendson L Church PT Banihani R. Follow-up care of the extremely preterm infant after discharge from the neonatal intensive care unit. Paediatr Child Health. (2022) 27:359–71. 10.1093/pch/pxac058 36200103 PMC9528778

[29] Eriksson MA Westerlund J Hedvall Å Åmark P Gillberg C. Fernell E. Medical conditions affect the outcome of early intervention in preschool children with autism spectrum disorders. Eur Child Adolesc Psychiatry. (2013) 22:23–33. 10.1007/s00787-012-0312-7 22836733

[30] van Rijn S. A review of neurocognitive functioning and risk for psychopathology in sex chromosome trisomy (47, XXY, 47,XXX, 47, XYY). Curr Opin Psychiatry. (2019) 32:79–84. 10.1097/YCO.0000000000000471 30689602 PMC6687415

[31] Rasmussen P Börjesson O Wentz E Gillberg C. Autistic disorders in Down syndrome: background factors and clinical correlates. Dev Med Child Neurol. (2001) 43:750–4. 10.1017/S0012162201001372 11730149

[32] Oxelgren UW Myrelid Å Annerén G Ekstam B Göransson C Holmbom A. Prevalence of autism and attention-deficit-hyperactivity disorder in Down syndrome: a population-based study. Dev Med Child Neurol. (2017) 59:276–83. 10.1111/dmcn.13217 27503703

[33] Leblond CS Le TL Malesys S Cliquet F Tabet AC Delorme R. Operative list of genes associated with autism and neurodevelopmental disorders based on database review. Mol Cell Neurosci. (2021) 113:103623. 10.1016/j.mcn.2021.103623 33932580

[34] Leblond CS Cliquet F Carton C Huguet G Mathieu A Kergrohen T. Both rare and common genetic variants contribute to autism in the Faroe Islands. NPJ Genom Med. (2019) 4:1. 10.1038/s41525-018-0075-2 30675382 PMC6341098

[35] Lichtenstein P Carlström E Råstam M Gillberg C Anckarsäter H. The genetics of autism spectrum disorders and related neuropsychiatric disorders in childhood. Am J Psychiatry. (2010) 167:1357–63. 10.1176/appi.ajp.2010.10020223 20686188

[36] Winston M Nayar K Hogan AL Barstein J La Valle C Sharp K. Physiological regulation and social-emotional processing in female carriers of the FMR1 premutation. Physiol Behav. (2020) 214:112746. 10.1016/j.physbeh.2019.112746 31765665 PMC6992413

[37] Maruani A Huguet G Beggiato A ElMaleh M Toro R Leblond CS. 11q24.2-25 micro-rearrangements in autism spectrum disorders: Relation to brain structures. Am J Med Genet A. (2015) 167A:3019–30. 10.1002/ajmg.a.37345 26334118

[38] Velinov M. Genomic copy number variations in the autism clinic-work in progress. Front Cell Neurosci. (2019) 13:57. 10.3389/fncel.2019.00057 30837845 PMC6389619

[39] Niklasson L Rasmussen P Oskarsdóttir S Gillberg C. Chromosome 22q11 deletion syndrome (CATCH 22): neuropsychiatric and neuropsychological aspects. Dev Med Child Neurol. (2002) 44:44–50. 10.1017/S0012162201001645 11811651

[40] Landgren M Svensson L Strömland K. Andersson Grönlund M. Prenatal alcohol exposure and neurodevelopmental disorders in children adopted from Eastern Europe. Pediatrics. (2010) 125:e1178–85. 10.1542/peds.2009-0712 20385628

[41] Landgren V Svensson L Gyllencreutz E Aring E Grönlund MA Landgren M. Fetal alcohol spectrum disorders from childhood to adulthood: a Swedish population-based naturalistic cohort study of adoptees from Eastern Europe. BMJ Open. (2019) 9:e032407. 10.1136/bmjopen-2019-032407 31666274 PMC6830611

[42] Gillberg IC Gillberg C Kopp S. Hypothyroidism and autism spectrum disorders. J Child Psychol Psychiatry. (1992) 33:531–42. 10.1111/j.1469-7610.1992.tb00889.x 1577897

[43] Bowden SA Goldis M. “Congenital Hypothyroidism,” In: StatPearls Treasure Island (FL): StatPearls Publishing (2022). 32644339

[44] Engman ML Sundin M Miniscalco C Westerlund J Lewensohn-Fuchs I Gillberg C. Prenatal acquired cytomegalovirus infection should be considered in children with autism. Acta Paediatr. (2015) 104:792–5. 10.1111/apa.13032 25900322

[45] Warrier V Zhang X Reed P Havdahl A Moore TM Cliquet F. Genetic correlates of phenotypic heterogeneity in autism. Nat Genet. (2022) 54:1–12. 10.1038/s41588-022-01072-5 35654973 PMC9470531

[46] Johnson S Marlow N. Growing up after extremely preterm birth: lifespan mental health outcomes. Semin Fetal Neonatal Med. (2014) 19:97–104. 10.1016/j.siny.2013.11.004 24290907

[47] Morsing E Lundgren P Hård AL Rakow A Hellström-Westas L Jacobson L. Neurodevelopmental disorders and somatic diagnoses in a national cohort of children born before 24 weeks of gestation. Acta Paediatr. (2022) 111:1167–75. 10.1111/apa.16316 35318709 PMC9454084

[48] Howlin P Magiati I Charman T. Systematic review of early intensive behavioral interventions for children with autism. Am J Intellect Dev Disabil. (2009) 114:23–41. 10.1352/2009.114:23-41 19143460

[49] Reichow B Hume K Barton EE Boyd BA. Early intensive behavioral intervention (EIBI) for young children with autism spectrum disorders (ASD). Cochrane Database Syst Rev. (2018) 5:CD009260. 10.1002/14651858.CD009260.pub3 29742275 PMC6494600

[50] Franz L Goodwin CD Rieder A Matheis M Damiano DL. Early intervention for very young children with or at high likelihood for autism spectrum disorder: an overview of reviews. Dev Med Child Neurol. (2022) 64:1063–76. 10.1111/dmcn.15258 35582893 PMC9339513

[51] Hedvall Å Westerlund J Fernell E Norrelgen F Kjellmer L Olsson MB. Preschoolers with autism spectrum disorder followed for 2 years: those who gained and those who lost the most in terms of adaptive functioning outcome. J Autism Dev Disord. (2015) 45:3624–33. 10.1007/s10803-015-2509-3 26123008

[52] FDA Approves Risperdal(R) for Treatment of Irritability Associated With Autistic Disorder. Available online at: https://www.biospace.com/article/releases/fda-approves-risperdal-r-for-treatment-of-irritability-associated-with-autistic-disorder-/ (accessed January 12, 2023).

[53] Tyzio R Nardou R Ferrari DC Tsintsadze T Shahrokhi A Eftekhari S. Oxytocin-mediated GABA inhibition during delivery attenuates autism pathogenesis in rodent offspring. Science. (2014) 343:675–9. 10.1126/science.1247190 24503856

[54] Lemonnier E Ben-Ari Y. The diuretic bumetanide decreases autistic behaviour in five infants treated during 3 months with no side effects. Acta Paediatr. (2010) 99:1885–8. 10.1111/j.1651-2227.2010.01933.x 20608900

[55] Rheims S Minlebaev M Ivanov A Represa A Khazipov R Holmes GL. Excitatory GABA in rodent developing neocortex *in vitro*. Neurophysiol. (2008) 100:609–19. 10.1152/jn.90402.2008 18497364

[56] Zhang L Huang CC Dai Y Luo Q Ji Y Wang K Deng S. Symptom improvement in children with autism spectrum disorder following bumetanide administration is associated with decreased GABA/glutamate ratios. Transl Psychiatry. (2020) 10:9. 10.1038/s41398-020-0747-4 32066666 PMC7026137

[57] Lemonnier E Villeneuve N Sonie S Serret S Rosier A Roue M. Effects of bumetanide on neurobehavioral function in children and adolescents with autism spectrum disorders. Transl Psychiatry. (2017) 7:e1056. 10.1038/tp.2017.10 28291262 PMC5416661

[58] James BJ Gales MA Gales BJ. Bumetanide for autism spectrum disorder in children: a review of randomized controlled trials. Ann Pharmacother. (2019) 53:537–44. 10.1177/1060028018817304 30501497

[59] Sprengers JJ van Andel DM Zuithoff NPA Keijzer-Veen MG Schulp AJA Scheepers FE. Bumetanide for core symptoms of autism spectrum disorder (BAMBI): a single center, double-blinded, participant-randomized, placebo-controlled, phase-2 superiority trial. J Am Acad Child Adolesc Psychiatry. (2021) 60:865–76. 10.1016/j.jaac.2020.07.888 32730977

